# Gender-typical olfactory regulation of sexual behavior in goldfish

**DOI:** 10.3389/fnins.2014.00091

**Published:** 2014-04-30

**Authors:** Yutaro Kawaguchi, Akira Nagaoka, Asana Kitami, Tomomi Mitsuhashi, Youichi Hayakawa, Makito Kobayashi

**Affiliations:** Department of Life Science, International Christian UniversityMitaka, Tokyo, Japan

**Keywords:** goldfish, sexual behavior, olfaction, olfactory tract, olfactory bulb, sex pheromone, prostaglandin

## Abstract

It is known that olfaction is essential for the occurrence of sexual behavior in male goldfish. Sex pheromones from ovulatory females elicit male sexual behavior, chasing, and sperm releasing act. In female goldfish, ovarian prostaglandin F2α (PGF) elicits female sexual behavior, egg releasing act. It has been considered that olfaction does not affect sexual behavior in female goldfish. In the present study, we re-examined the involvement of olfaction in sexual behavior of female goldfish. Olfaction was blocked in male and female goldfish by two methods: nasal occlusion (NO) which blocks the reception of olfactants, and olfactory tract section (OTX) which blocks transmission of olfactory information from the olfactory bulb to the telencephalon. Sexual behavior of goldfish was induced by administration of PGF to females, an established method for inducing goldfish sexual behavior in both sexes. Sexual behavior in males was suppressed by NO and OTX as previously reported because of lack of pheromone stimulation. In females, NO suppressed sexual behavior but OTX did not affect the occurrence of sexual behavior. Females treated with both NO and OTX performed sexual behavior normally. These results indicate that olfaction is essential in female goldfish to perform sexual behavior as in males but in a different manner. The lack of olfaction in males causes lack of pheromonal stimulation, resulting in no behavior elicited. Whereas the results of female experiments suggest that lack of olfaction in females causes strong inhibition of sexual behavior mediated by the olfactory pathway. Olfactory tract section is considered to block the pathway and remove this inhibition, resulting in the resumption of the behavior. By subtract sectioning of the olfactory tract, it was found that this inhibition was mediated by the medial olfactory tracts, not the lateral olfactory tracts. Thus, it is concluded that goldfish has gender-typical olfactory regulation for sexual behavior.

## Introduction

In fishes, olfaction is one of the important senses for their life cycle activities such as feeding, avoiding predation, and reproduction (Hamdani et al., 2000, 2001; Weltzien et al., 2003; Zielinski and Hara, 2007). It has been known that many teleost fishes employ sex pheromones to coordinate their reproductive activity with successful fertilization of gametes (Stacey and Sorensen, 2006; Stacey, 2011). These sex pheromones released from a signaler transmit information on sex and sexual maturity of the signaler to a receiver. Since most of fish sex pheromones studied are from female to male, the involvement of olfaction in reproductive activity has been intensively studied in male fish, and few studies are conducted on the involvement of olfaction in female reproduction (Sorensen et al., 2005; Lastein et al., 2006; Appelt and Sorensen, 2007; Stacey, 2011; Hayakawa et al., 2012).

Goldfish *Carassius auratus* is a small cyprinid species and intensively used for environmental and physiological studies of behavior. As a result, hormonal, pheromonal, and neural regulation of sexual behavior in goldfish is probably the best understood among fish species (Stacey and Sorensen, 2006; Munakata and Kobayashi, 2010; Stacey, 2011; Kobayashi et al., 2013). Female sexual behavior (egg releasing act) is induced by prostaglandin F2α (PGF) produced in the ovary at the time of ovulation. PGF and its metabolites are released into the water as sex pheromones which stimulate male sexual behavior (chasing and sperm releasing act). For the occurrence of sexual behavior, estrogens are not required by females (Kobayashi and Stacey, 1993), but androgen is considered to be essential for males (Stacey and Kobayashi, 1996). When PGF is injected into non-ovulatory females, these fish are induced to perform female sexual behavior with normal males within several minutes after the PGF injection although no egg release is accompanied in this case (Stacey and Kyle, 1983). Thus, using PGF, sexual behavior can be induced easily in goldfish pairs.

Anatomy of olfactory system of goldfish and crucian carp *Carassius carassius*, closely related species of goldfish, is well studied (Von Bartheld et al., 1984; Hamdani et al., 2000, 2001; Weltzien et al., 2003; Stacey and Sorensen, 2006), which enables surgical block of olfaction in goldfish. In the present study, we examined involvement of olfaction in sexual behavior in male and female goldfish by two methods of olfactory blockage. The first method is occlusion of nasal cavity with glue which blocks reception of olfactants. The second method is olfactory tract section. Since the olfactory bulbs of goldfish are the pedunculated type with elongated olfactory tracts, olfactory information from the olfactory bulbs to the telencephalon can be easily blocked by sectioning olfactory tracts (Stacey and Kyle, 1983; Von Bartheld et al., 1984; Kobayashi et al., 1986, 1994). Using these two methods, we compared the effects of olfactory blockage on sexual behavior between male and female goldfish.

## Materials and methods

### Fish

Goldfish *Carassius auratus* were obtained from a local dealer in Saitama Prefecture, Japan. Fish were kept in 800-L stock tanks maintained at 20°C under 16L/8D photoperiod. Fish were freely fed with commercial goldfish feed once a day. It is known that gonadal maturity of goldfish is generally maintained under these environmental conditions. Most of the stock males were spermiating and had tubercles on their pectoral fins (male secondary sexual characteristic), and females were found to have vitellogenic oocytes in the ovary when randomly sampled and dissected. Fish weighing 14–30 g were used for the experiments. The handling of fish in the present study was endorsed by Animal Experimentation Committee of International Christian University.

### Sexual behavior of goldfish

Natural and prostaglandin (PG)-induced spawning (sexual) behaviors are well documented in goldfish (Kobayashi et al., 2002; Munakata and Kobayashi, 2010). In brief, during natural spawning, ovulated females produce PGF in the ovary, and this PGF acts on the brain and triggers the female spawning act (egg releasing act) in the females. PGF and its metabolites are released into the water as sex pheromones which trigger male spawning behavior that are characterized as chasing and culminating in sperm release (i.e., male spawning act). Male chasing is persistent and interspersed with the spawning acts. Spawning acts (complete spawning act) are initiated by the entry of an ovulated female into the floating aquatic vegetation near the surface of the water and where the male follows the female. The female and the male turn on their sides and swim quickly through the vegetation, releasing eggs and sperm. The male always positions itself underneath and in contact with the female during this act. Then, they flip their tails to mix spawned eggs and sperm. Released eggs are characteristically sticky and quickly adhere to the vegetation. Female spawning will continue until most of her ovulated eggs are released, and this may involve hundreds or more spawning acts over several hours. Another type of spawning act of goldfish is called an incomplete spawning act. An incomplete (attempted) spawning act is similar to a complete spawning act except that the fish leave the vegetation without performing gamete release and tail flipping. In the present study, we considered both complete and incomplete spawning acts as normal behavior and were counted equally.

Female goldfish injected with PGF are induced to perform the female spawning act as do ovulated females with sexually mature males, although eggs are not released. Males do not distinguish between ovulated and PG-injected females. In the present paper, the female spawning act (egg releasing act) is referred to as female sexual behavior, and the male spawning act (sperm releasing act) is referred to as male sexual behavior in goldfish.

### Behavior experiments

For the behavior experiments, fish were transferred from stock tanks to 60-L glass acclimating aquaria and kept at 20°C under 16L/8D photoperiod over the course of 1–10 days. Each behavior test was conducted in 60-L glass observation aquaria provided with artificial floating vegetation made of acrylic yarn, gravel, and an aerated box filter and water temperature maintained at 20°C and a 16L/8D photoperiod.

Prostaglandin F2α (PGF) (Panaseran Hi, Meiji Seika, Tokyo, Japan) was intramuscularly injected into females with a microsyringe for the induction of sexual behavior of goldfish just before each sexual behavior test. PGF was injected into experimental females at a dose of 0.1 μg/0.1 μL saline/g body weight for the female behavior experiments, and to partner females at a dose of 10 μg/2.0 μL saline/fish for the male behavior experiments (Stacey and Kyle, 1983; Saoshiro et al., 2013).

For male sexual behavior tests, experimental males and PG-injected partner females were paired in each observation aquarium and sexual behavior (total of complete and incomplete spawning acts) was counted for 60 or 90 min (Stacey and Kyle, 1983). For female behavior tests, PG-injected experimental females and partner males were paired in each observation aquarium and sexual behavior (total of complete and incomplete spawning acts) was counted for 60 or 90 min (Stacey and Kyle, 1983). Pre-treatment tests were conducted on Day 1 and Post-treatment tests were conducted on Day 3 (Table 1). Each experimental fish was paired with the same partner fish in pre- and post-treatment tests. Fish received no treatment or behavior test on Day 2 for the recovery from the treatment and behavior test. Experimental fish and partner fish were kept in 60-L glass acclimating aquaria between the tests.

**Table 1:** **Experimental design of sexual behavior in goldfish**.

	**Day 1**	**Day 3**
Experiment 1	Sex behavior test	Nasal occlusion and sex behavior test
Experiment 2	Sex behavior test and OTX	Sex behavior test
Experiment 3	Sex behavior test	Nasal occlusion and sexual behavior test
Experiment 4	Sex behavior test and OTX	Sex behavior test
Experiment 5	Nasal occlusion, sex behavior test, and OTX	Nasal occlusion and sexual behavior test
Experiment 6	Sex behavior test and subtract section	Nasal occlusion and sexual behavior test
Experiment 7	Sex behavior test and subtract section	Nasal occlusion and sex behavior test

Fish received no treatment or behavior test on Day 2. OTX, olfactory tract section.

### Blocakge of olfaction

For the blockage of olfaction, two methods were employed in this study: nasal occlusion which blocks reception of olfactants, and olfactory tract section (OTX) which blocks transmission of olfactory information from the olfactory bulb to the telencephalon where the neural center regulating sexual behavior is located (Kyle et al., 1982; Koyama et al., 1985).

Nasal occlusion was carried out according to the method previously reported (Partridge et al., 1976). Fish were anaesthetized by immersion in 0.02% tricaine methanesulfonate (Sigma-Aldrich, St. Louis, MO, USA) solution. The nasal cavities were occluded with denture fix (Shin-Poligrip-SA Mutenka, Earth Chemical, Tokyo, Japan). Then, fish were kept in dechlorinated water for 10–20 min for recovery from anesthesia before behavior tests.

For the complete blockage of olfaction, the nasal cavities of both sides were bilaterally occluded. As one of the controls, one side of nasal cavity was unilaterally occluded. As another control, fish were treated in the same manner without occlusion of the nasal cavities (intact). This method of occlusion was effective in blocking olfaction for 70–80 min since denture fix used in the present study is water-soluble and some bilaterally occluded males started male sexual behavior with PG-injected females around this time, probably due to the leakage of the water to the olfactory epithelium. Therefore, observation period of experiment using this method was set to 60 min.

It is well established that PG pheromone acts on the olfactory epithelium of sexually mature male goldfish and elicits chasing (courtship) of the males (Stacey and Sorensen, 2006). To assess the completeness of nasal occlusion, we examined the suppression of chasing of male goldfish in the presence of the PG-injected females. We tried other methods of olfactory blockage, such as cauterization of olfactory epithelium and application of silver nitrate solution on the epithelium, but these techniques were not successful (Kobayashi, unpublished data) and nasal occlusion was effective for 70–80 min. The methods that are effective for the olfactory blockage in males, nasal occlusion and olfactory tract section, were applied to the experiments of females in the present study.

Olfactory tract section was carried out according to the method previously reported (Stacey and Kyle, 1983; Kobayashi et al., 1986, 1994). Fish were anaesthetized with a 0.02% tricaine methanesulfonate solution. A four-sided flap was cut in the frontal bone using a disc saw in order to expose a pair of the olfactory tracts. After removal of a square flap of the frontal bone, the tracts were bilaterally or unilaterally sectioned at two places with Wecker's scissors, and the resultant sections were removed (OTX). Olfactory tracts were subdivided surgically into a lateral olfactory tract (LOT) and a medial olfactory tract (MOT) that can be further separated into lateral medial-olfactory tract (lMOT) and medial medial-olfactory tract (mMOT). Specific subtract section was also carried out. The cavity resulting from operation was filled with gelatin sponge (Spongel, Yamanouchi Pharamaceutical Co. Tokyo, Japan). Sham operations were performed in the same manner without cutting the olfactory tracts.

### Effects of nasal occlusion on male sexual behavior (experiment 1)

In this experiment, involvement of olfaction in sexual behavior in male goldfish was examined by nasal occlusion which blocks the reception of sex pheromone from partner female. On Day 1, experimental males were paired with PG-injected partner females in observation aquaria, and male sexual behavior (sperm releasing act) was counted over a period of 60 min (pre-treatment test). On Day 3, experimental males were paired with PG-injected partner females after one of the following three treatments, bilateral nasal occlusion, unilateral nasal occlusion, and intact, and male sexual behavior was observed for 60 min (post-treatment test).

### Effects of olfactory tract section on male sexual behavior (experiment 2)

In this experiment, involvement of olfaction in sexual behavior in male goldfish was examined by OTX which blocks the transmission of olfactory information from the olfactory bulb to the telencephalon. On Day 1, experimental males were paired with PG-injected partner females in observation aquaria, and male sexual behavior was counted over a period of 90 min (pre-treatment test). After the behavior tests, experimental males received either OTX or sham operation. On Day 3, experimental males were paired with PG-injected partner females, and male sexual behavior was observed for 90 min (post-treatment test).

### Effects of nasal occlusion on female sexual behavior (experiment 3)

In this experiment, involvement of olfaction in sexual behavior in female goldfish was examined by nasal occlusion which blocks the reception of olfactants from the environment. On Day 1, experimental females were injected with PGF, paired with partner males in observation aquaria, and female sexual behavior (egg releasing act) was counted over a period of 60 min (pre-treatment test). On Day 3, experimental females were injected with PGF, paired with partner males after one of the following three treatments, bilateral nasal occlusion, unilateral nasal occlusion, and intact, and female sexual behavior was observed for 60 min (post-treatment test).

### Effects of olfactory tract section on female sexual behavior (experiment 4)

In this experiment, involvement of olfaction in sexual behavior in female goldfish was examined by OTX. On Day 1, experimental females were injected with PGF, paired with partner males in observation aquaria, and female sexual behavior was counted over a period of 90 min (pre-treatment test). After the behavior tests, experimental females received either OTX or sham operation. On Day 3, experimental females were injected with PGF, paired with partner males, and female sexual behavior was observed for 90 min (post-treatment test).

### Effects of nasal occlusion and olfactory tract section on female sexual behavior (experiment 5)

In this experiment, effects of simultaneous treatments of nasal occlusion and OTX were examined in female goldfish. On Day 1, experimental females were injected with PGF, paired with partner males in observation aquaria after bilateral nasal occlusion, and female sexual behavior was counted over a period of 60 min (pre-treatment test). After the behavior tests, experimental females received either OTX or sham operation. On Day 3, experimental females were injected with PGF, paired with partner males after bilateral treatment of nasal occlusion, and female sexual behavior was observed for 60 min (post-treatment test).

### Effects of LOT and MOT section on female sexual behavior (experiment 6)

In this experiment, effects of LOT and MOT section on sexual behavior were examined in female goldfish. On Day 1, experimental females were injected with PGF, paired with partner males in observation aquaria, and female sexual behavior was counted over a period of 60 min (pre-treatment test). After the behavior tests, experimental females received either LOT section or MOT section. On Day 3, experimental females were injected with PGF, paired with partner males after bilateral nasal occlusion, and female sexual behavior was observed for 60 min (post-treatment test).

### Effects of lateral MOT and medial MOT section on female sexual behavior (experiment 7)

In this experiment, effects of lMOT and mMOT section on sexual behavior were examined in female goldfish. On Day 1, experimental females were injected with PGF, paired with partner males in observation aquaria, and female sexual behavior was counted over a period of 60 min (pre-treatment test). After the behavior tests, experimental females received either lMOT section or mMOT section. On Day 3, experimental females were injected with PGF, paired with partner males after bilateral nasal occlusion, and female sexual behavior was observed for 60 min (post-treatment test).

### Statistics

Data among groups were statistically compared by ANOVA and Newman-Keuls test. Data between groups were statistically compared by Mann-Whitney *U*-test. Data within a group were statistically compared by paired *t*-test. Level of significance was 0.05 for all statistical tests. Statistical test could not be applied to some data of groups because mean values were zero or close to zero with very low deviation.

## Results

### Effects of nasal occlusion on male sexual behavior (experiment 1)

**Figure 1:**
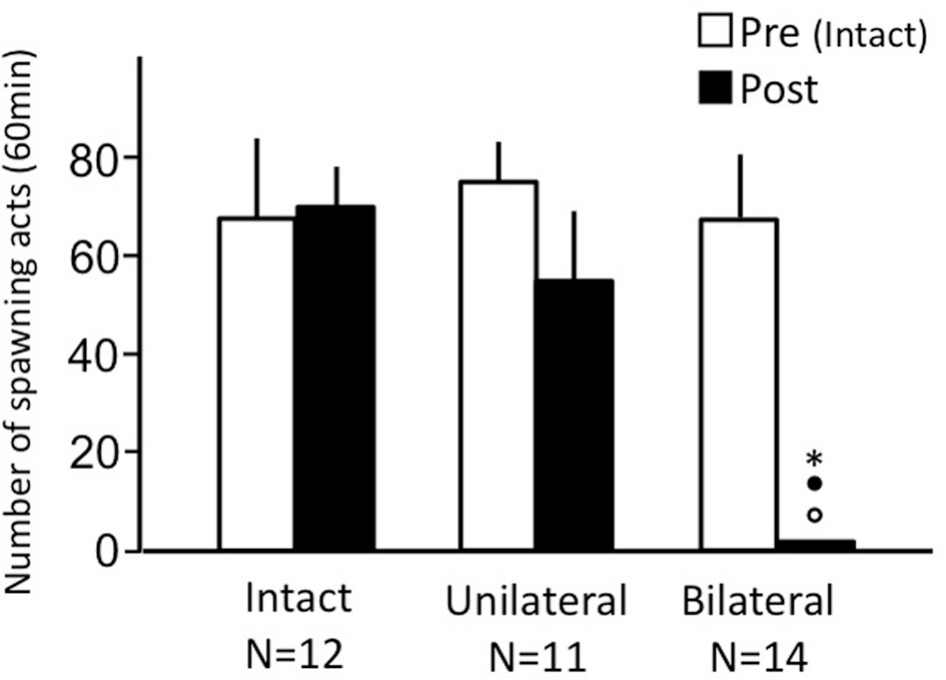
**Effects of nasal occlusion on male sexual behavior in male goldfish**. Pre, Pre-treatment (open column). Post, post-treatment (solid column). Each column represents the mean and s.e.m. Difference compared to pre-treatment (asterisk), compared to intact fish (solid circle), and compared to unilaterally occluded fish (open circle). Level of significance, *p* < 0.05.

Intact fish and unilaterally occluded fish actively performed male sexual behavior in pre- and post-treatment tests on Day 1 and Day 3, respectively (Figure 1). Levels of sexual behavior in these fish showed no change between Day 1 and Day 3 (*p* > 0.05). Level of sexual behavior in male goldfish significantly decreased after bilateral nasal occlusion (*p* < 0.05, compared to pre-treatment level and to levels of intact and unilaterally occluded fish of Day 3).

### Effects of olfactory tract section on male sexual behavior (experiment 2)

**Figure 2:**
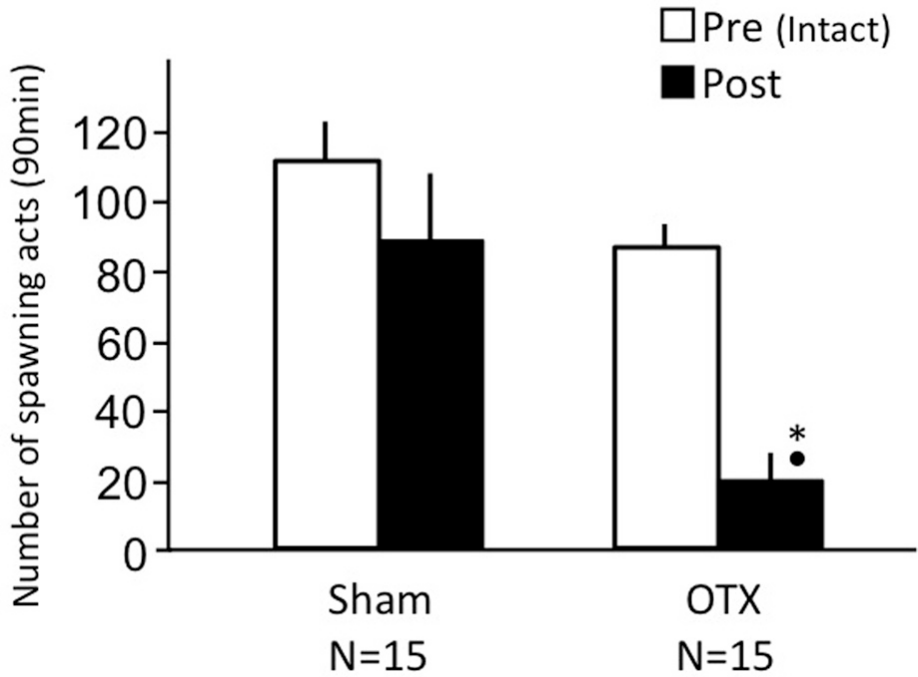
**Effects of olfactory tract section on male sexual behavior in male goldfish**. Pre, Pre-treatment (open column). Post, post-treatment (solid column). Each column represents the mean and s.e.m. Difference compared to pre-treatment (asterisk), compared to sham-operated fish (solid circle). Level of significance, *p* < 0.05.

Sham-operated fish actively performed male sexual behavior in pre- and post-treatment tests on Day 1 and Day 3, respectively (Figure 2). Levels of sexual behavior in sham-operated fish showed no change between Day 1 and Day 3 (*p* > 0.05). Level of sexual behavior in male goldfish significantly decreased after OTX (*p* < 0.05, compared to pre-treatment level and to level of sham operated fish of Day 3).

### Effects of nasal occlusion on female sexual behavior (experiment 3)

**Figure 3:**
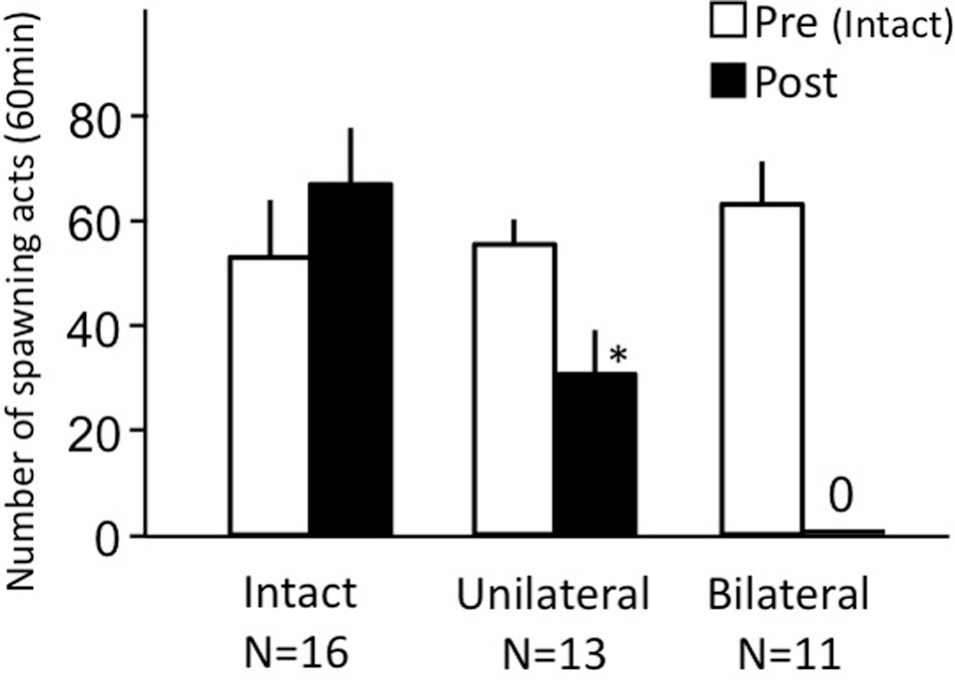
**Effects of nasal occlusion on female sexual behavior in female goldfish**. Pre, Pre-treatment (open column). Post, post-treatment (solid column). Each column represents the mean and s.e.m. Difference compared to pre-treatment (asterisk), *p* < 0.05.

Intact fish actively performed female sexual behavior in pre- and post-treatment tests on Day 1 and Day 3, respectively (Figure 3). Levels of sexual behavior in intact fish showed no change between Day 1 and Day 3 (*p* > 0.05). Level of sexual behavior in female goldfish significantly decreased after unilateral nasal occlusion (*p* < 0.05 compared to pre-treatment level). Female goldfish of bilateral occlusion showed no sexual behavior on Day 3.

### Effects of olfactory tract section on female sexual behavior (experiment 4)

**Figure 4:**
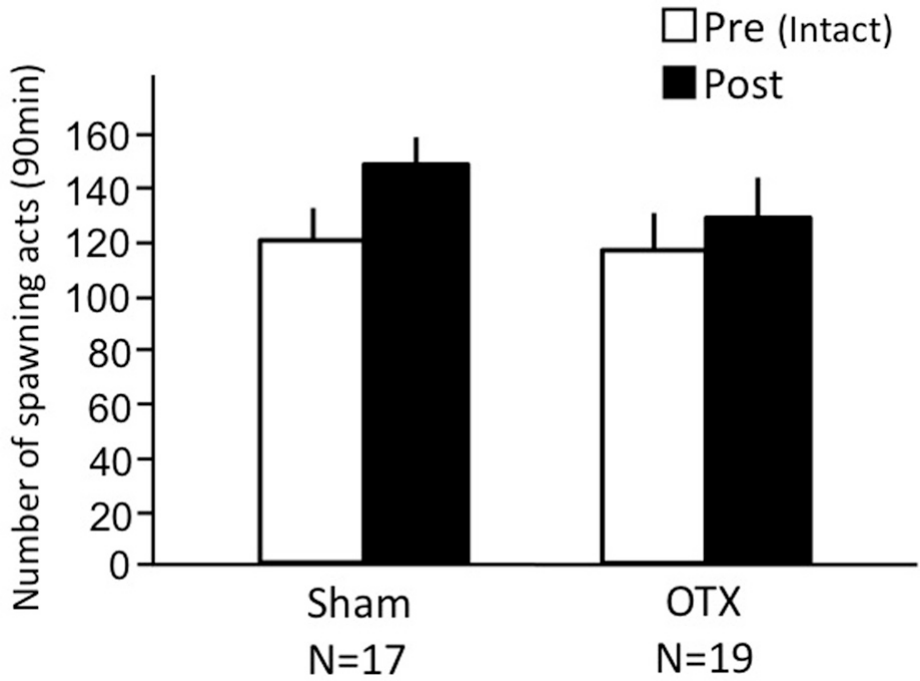
**Effects of olfactory tract section on female sexual behavior in female goldfish**. Pre, Pre-treatment (open column). Post, post-treatment (solid column). Each column represents the mean and s.e.m.

Both sham-operated fish and OTX fish actively performed female sexual behavior in pre- and post-treatment tests on Day 1 and Day 3, respectively (Figure 4). Levels of sexual behavior in these fish showed no change between Day 1 and Day 3 (*p* > 0.05).

### Effects of nasal occlusion and olfactory tract section on female sexual behavior (experiment 5)

**Figure 5:**
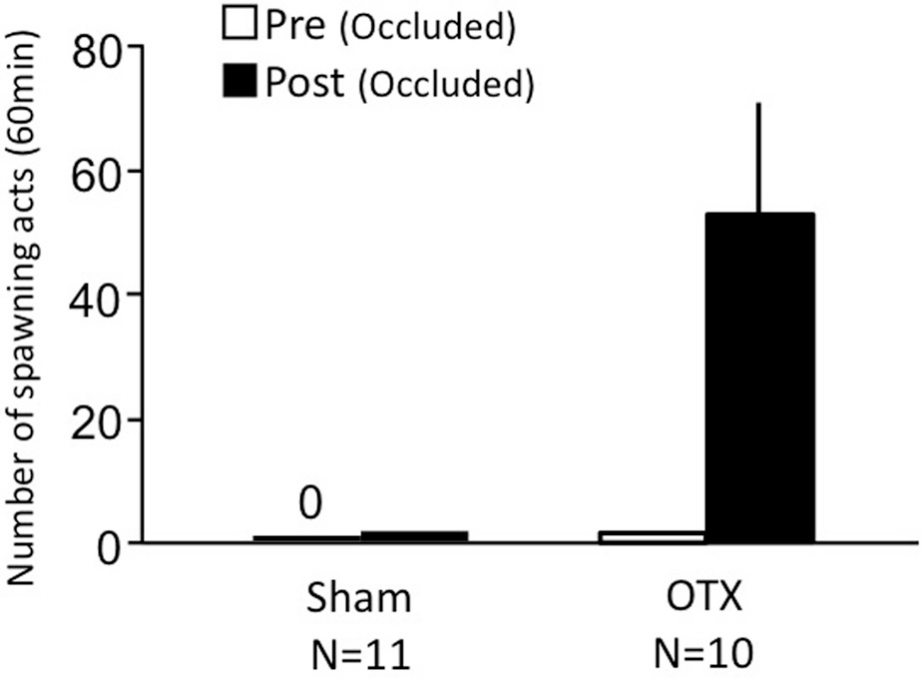
**Effects of nasal occlusion and olfactory tract section on female sexual behavior in female goldfish**. Pre, Pre-treatment (bilaterally occluded). Post, post-treatment (bilaterally occluded and either sham-operated or olfactory-tract sectioned). Each column represents the mean and s.e.m.

Females with bilateral nasal occlusion showed almost no sexual behavior on Day 1 (Figure 5). Sham-operated fish with bilateral occlusion showed almost no sexual behavior on Day 3. OTX fish with bilateral occlusion actively performed sexual behavior on Day 3.

### Effects of LOT and MOT section on female sexual behavior (experiment 6)

**Figure 6:**
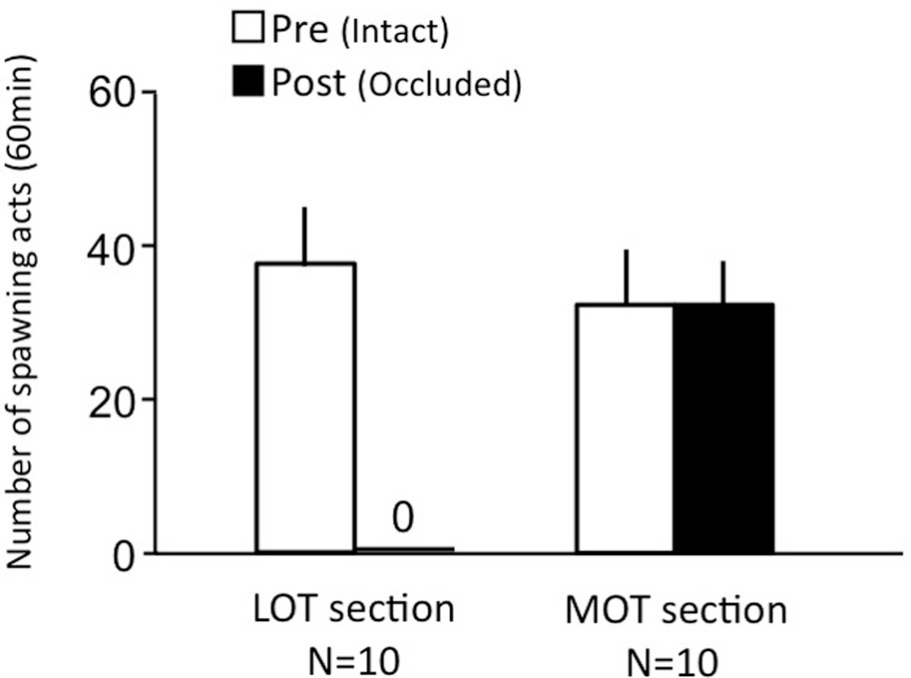
**Effects of olfactory subtract section on female sexual behavior in female goldfish**. LOT section, Lateral olfactory tracts were sectioned. MOT, Medial olfactory tracts were sectioned. Pre-treatment (open column). Post, post-treatment (solid column). Each column represents the mean and s.e.m.

Females before treatment actively performed sexual behavior on Day 1 (Figure 6). LOT-sectioned fish with bilateral nasal occlusion showed no sexual behavior on Day 3. MOT-sectioned fish with bilateral nasal occlusion actively performed sexual behavior on Day 3.

### Effects of lateral MOT and medial MOT section on female sexual behavior (experiment 7)

**Figure 7:**
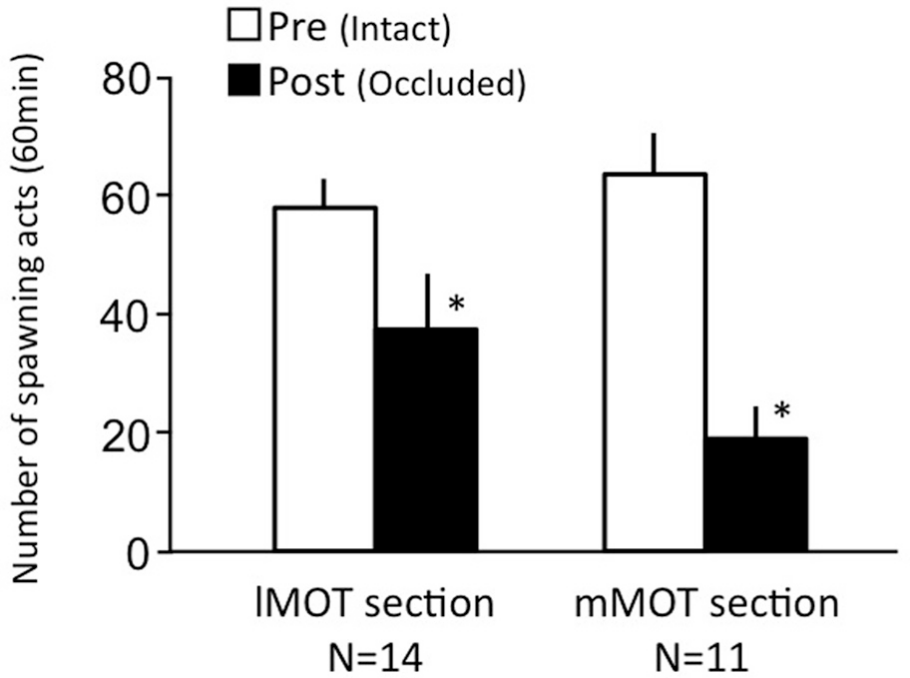
**Effects of section of medial olfactory tract components on female sexual behavior in female goldfish**. lMOT section, lateral medial-olfactory tract section. mMOT section, medial medial-olfactory tract section. Pre-treatment (open column). Post, post-treatment (solid column, bilaterally occluded and either lMOT sectioned or mMOT sectioned). Each column represents the mean and s.e.m. Difference compared to pre-treatment (asterisk). Level of significance, *p* < 0.05.

Females before treatment actively performed sexual behavior on Day 1 (Figure 7). Levels of sexual behavior in lMOT-sectioned fish and mMOT-sectioned fish with bilateral nasal occlusion significantly decreased on Day 3 (*p* < 0.05, compared to pretreatment level).

## Discussion

The present study indicates that olfaction is the key prerequisite for the occurrence of sexual behavior both in male and female goldfish, but its regulation for sexual behavior is different between male and female. In male goldfish, blockage of olfaction by nasal occlusion and OTX severely reduced activity of sexual behavior as previously reported (Partridge et al., 1976; Stacey and Kyle, 1983) because of lack of pheromonal stimulation. Since males with unilateral nasal occlusion actively performed sexual behavior as did intact males, chemical or physical damage of nasal occlusion on fish is considered to be negligible. Some males with bilateral nasal occlusion and OTX showed weak male sexual behavior. These males did not show vigorous chasing that intact males normally do to PG-injected partner females, and sexual behavior of these males occurred mostly when the partner females rose into the aquatic vegetation near these anosmic males. It is considered that anosmic males sometimes show sexual behavior by visual stimulus from females (Norm Stacey and Peter Sorensen, personal communication), and not due to incompleteness of nasal occlusion and OTX. Thus, we examined the involvement of olfaction in female sexual behavior using the same methods that were used for the experiments for males.

Female sexual behavior was strongly suppressed by bilateral nasal occlusion which blocks the reception of olfactants even under the stimulation of PGF. Level of sexual behavior was also reduced by unilateral nasal occlusion. The results of these experiments indicate that the olfaction is essential for the female sexual behavior to occur. However, OTX fish performed female sexual behavior after PG-injection as reported previously (Stacey and Kyle, 1983) although these females were unable to receive olfactory information from the olfactory bulbs. Since OTX did not affect the occurrence of female sexual behavior in PG-injected female goldfish in the previous study (Stacey and Kyle, 1983), it has been considered that olfaction is not essential for the female sexual behavior in goldfish. Nevertheless, nasal occlusion suppressed the female sexual behavior. Results of experiments of nasal occlusion and OTX look contradictory, but one of the possible interpretations we propose is as follows. Blockage of olfaction in females exerts strong inhibition of sexual behavior mediated by the olfactory pathway from the olfactory epithelium to the telencephalon via the olfactory bulb and the olfactory tract even in the presence of PGF stimulation. When females were treated with nasal occlusion and OTX simultaneously, the fish actively performed sexual behavior. Therefore, it is considered that OTX blocked the pathway and removed the inhibitory signaling for the behavior. Reduction of female sexual behavior in unilaterally occluded female may be caused by some inhibition from the occluded side of olfactory epithelium unlike the case of unilaterally occluded male goldfish. Sexual behavior of unilaterally occluded male can receive enough amount of pheromonal stimulation by one side of olfactory epithelium and perform full range of sexual behavior. We further examined the pathway of the inhibitory signaling in female goldfish by olfactory subtract section. Section of LOT did not remove the inhibition of sexual behavior in bilaterally occluded females, but by MOT section, bilaterally occluded females performed sexual behavior, indicating that the inhibition in the olfactory pathway is transmitted via MOT. When lMOT and mMOT was individually sectioned, bilateral nasal occlusion caused partial inhibition of female sexual behavior, suggesting that both lMOT and mMOT are involved in mediating the inhibitory signaling.

It is known that MOT mediates pheromonal information and that LOT mediates information of food odors in male and female goldfish and crucian carp (Stacey and Kyle, 1983; Hamdani et al., 2001; Weltzien et al., 2003). It is interesting that MOT is involved in regulation of sexual behavior both in male and female goldfish although its function is different, stimulatory in males and inhibitory in females. A clear functional difference of the olfactory bulb between male and female is reported in crucian carp. Electrophysiological study showed that the olfactory bulb of male carp discriminates sex pheromones but that of female does not (Lastein et al., 2006). It is suggested that these gender-typical function of pheromone detection and sexual behavior is developed by androgens since female fish start to perform male sexual behavior and male type gonadotropin secretion in response to sex pheromones in goldfish and crucian carp *Carassius auratus langsdorfii* (Stacey and Kobayashi, 1996; Kobayashi et al., 1997; Kobayashi and Nakanishi, 1999). It is also interesting to study how olfaction is involved in sexual behavior of females in other teleost species. Our recent study in medaka *Oryzias latipes* showed that male sexual behavior was severely suppressed by olfactory blockage (nasal occlusion) but female sexual behavior was not affected by nasal occlusion unlike female goldfish (Hayakawa et al., 2012).

The present study demonstrated the involvement of olfaction in female sexual behavior in goldfish. However, biological significance of olfactory regulation in female goldfish is not clarified. There are some possible explanations of female olfactory system for the regulation of sexual behavior. One of the possible functions could be detection of special odorants, such as sex pheromone from males. There are some reports that female sexual activity is stimulated by sex pheromones from males (Stacey and Sorensen, 2006; Stacey, 2011). In the case of female goldfish, PGF triggers female sexual behavior and a male sex pheromone may not be a trigger but could facilitate the behavior. It is known that a large amount of androstenedione is released from sexually mature male goldfish into the water (Sorensen et al., 2005) and that PG-injected female goldfish show rise into vegetation (initial movement of female spawning act) more frequently in the presence of sexually mature males than in the absence of males (Appelt and Sorensen, 2007). It is possible that androstendione from males function as a sex pheromone which stimulates the initiation of female sexual behavior.

Another possible explanation is that female goldfish searches appropriate site and substrate for oviposition using sense of olfaction as well as vision and tactility. Normally females, not males, decide the site of oviposition in goldfish, and female goldfish need to select appropriate oviposition substrate which secures development of embryo and also appropriate site of water quality. Female goldfish may use olfaction for examining water quality of the spawning site. When olfaction is blocked in female goldfish which fails to examine the water quality of the spawning area, sexual behavior is inhibited even if the fish is stimulated by PGF. Such hypothesis can be applied in male tilapia *Oreochromis niloticus* which makes nest for spawning (Uchida et al., 2005). Olfactory blockage by removal of olfactory rosettes suppressed nest-buliding behavior in the male tilapia although activity of sexual behavior (courtship to female) was unchanged. Female salmonid fishes are known to stop nest-digging behavior and following spawning behavior when pH of the water is slightly lowered even when other environmental conditions are suitable for spawning (Ikuta et al., 2003). It is possible that these females sense the acidity by olfaction. It is of interest to examine whether water quality change may affect sexual behavior in intact and OTX female goldfish.

In conclusion, the present study demonstrated the involvement of olfaction in female sexual behavior in goldfish although it has been considered that olfaction does not affect the behavior. When reception of olfactants is blocked, female sexual behavior is severely suppressed. It is considered that lack of olfaction exerts inhibition of the behavior mediated by the olfactory pathway. Olfactory tract section removes this inhibition resulting in resumption of sexual behavior. Biological significance of this inhibitory system of female individual is unknown. Further behavioral and ecological studies are necessary for better understanding of the regulation of sexual behavior by olfaction in fishes. Also, it is interesting to study the gender typical-olfactory function could be induced heterotypically in goldfish since heterotypical sexual behavior and hormone secretion could be induced in goldfish (Stacey and Kobayashi, 1996; Kobayashi et al., 1997) and crucian carp *C. auratus langsdorfii* (Kobayashi and Nakanishi, 1999) which are considered to have sexually bipotential brain (Kobayashi et al., 2013).

### Conflict of interest statement

The authors declare that the research was conducted in the absence of any commercial or financial relationships that could be construed as a potential conflict of interest.
